# Quantum algorithm for quicker clinical prognostic analysis: an application and experimental study using CT scan images of COVID-19 patients

**DOI:** 10.1186/s12911-021-01588-6

**Published:** 2021-07-30

**Authors:** Kinshuk Sengupta, Praveen Ranjan Srivastava

**Affiliations:** 1 Microsoft Corporation, New Delhi , India; 2 Department of Information System, Indian Institute of Management, Rohtak, India; 3 City Southern Bypass, Sunaria, Rohtak, Haryana 124010 India

**Keywords:** Medical imaging and analysis, Artificial intelligence, Quantum neural networks, Medical informatics

## Abstract

**Background:**

In medical diagnosis and clinical practice, diagnosing a disease early is crucial for accurate treatment, lessening the stress on the healthcare system. In medical imaging research, image processing techniques tend to be vital in analyzing and resolving diseases with a high degree of accuracy. This paper establishes a new image classification and segmentation method through simulation techniques, conducted over images of COVID-19 patients in India, introducing the use of Quantum Machine Learning (QML) in medical practice.

**Methods:**

This study establishes a prototype model for classifying COVID-19, comparing it with non-COVID pneumonia signals in Computed tomography (CT) images. The simulation work evaluates the usage of quantum machine learning algorithms, while assessing the efficacy for deep learning models for image classification problems, and thereby establishes performance quality that is required for improved prediction rate when dealing with complex clinical image data exhibiting high biases.

**Results:**

The study considers a novel algorithmic implementation leveraging quantum neural network (QNN). The proposed model outperformed the conventional deep learning models for specific classification task. The performance was evident because of the efficiency of quantum simulation and faster convergence property solving for an optimization problem for network training particularly for large-scale biased image classification task. The model run-time observed on quantum optimized hardware was 52 min, while on K80 GPU hardware it was 1 h 30 min for similar sample size. The simulation shows that QNN outperforms DNN, CNN, 2D CNN by more than 2.92% in gain in accuracy measure with an average recall of around 97.7%.

**Conclusion:**

The results suggest that quantum neural networks outperform in COVID-19 traits’ classification task, comparing to deep learning w.r.t model efficacy and training time. However, a further study needs to be conducted to evaluate implementation scenarios by integrating the model within medical devices.

## Background

In the clinical trial and drug discovery process, the role of statistical analytics and machine learning has been shown to be significant, especially in biological imaging and analysis, commonly used at various stages, from preclinical R&D to clinical trials, solving problems like sputum detection [1], image augmentation [2] and other applications, such as nucleus counting [3]. In the recent past, substantial research work have been proposed studying various classical machine learning and deep learning methods applied to an image that assists scientists and medical practitioners in analyzing and seeing inorganic growth or accumulation of tissues, cells, and subcellular components in CT scans, along with a more technology-oriented solution in the space of wearable technology [4] and tele-health care services to discover COVID-19 [5]. An example of detecting brain tumors through deep learning methods has been studied by researchers [6] and diverse COVID-19 diagnosis research work using deep learning and traditional machine learning methods as shown in Table 1. Currently, with evolving COVID-19 mutants it is now becoming extremely important to leverage faster and accurate solutions for clinical discovery, prompting therefore our study to understand the evolution in terms of offering medical imaging solutions for factor detection of mutant variants [7].

**Table 1:** Empirical research for detecting COVID-19 using deep learning^a^

Model proposed	Study	Dataset size	Training samples sufficiency	Model performance
MODE (Multi-objective differential evolution) based CNN	Singh et al. [47]	1000 + CT images	+ + +	Accuracy—90.6%
UNET + +	Chen et al. [44]	46,000 + CT images	+ + +	Accuracy—95.24% Sensitivity—100% Specificity—93.55%
Stacked Two CNN three dimensional for classification and VNET for Segmentation	Xu et al. [43]	19,000 + CT Images with COVID-19, 1175 healthy samples	+ + +	Accuracy—86.70%
COVNet + ResNet 50 for classification and U-Net for segmentation	Li et al. [35]	4000 + CT Samples	+ + +	Sensitivity—90.0% Specificity—96.0%
Transfer Learning + ResNet 50 for classification and UNet + + (3D) for segmentation	Jin et al. [10]	1100 + total samples with 730 positive samples	+ +	AUC—0.991 Sensitivity—97.4% Specificity—92.2%
Inception with Transfer Learning technique	Wang et al. [32]	450 + CT scans of confirmed COVID-19	+	Accuracy—82.9% Sensitivity—84.0% Specificity—80.5%
Neural Networks with ResNET 50, attention technique and Feature Pyramid Network	Song et al. [42]	750 + Images	+	Accuracy—86.0% F-Score—87.0% Sensitivity—93.0%
Deep Conv Net(2D) on ResNet-50 for classification and UNet for segmentation	Gozes et al. [41]	50 + patients’ samples	+	AUC—0.996 Sensitivity—98.2% Specificity—92.2%
VBNet neural network to Segment COVID-19 infection regions in CT scans	Shan et al. [13]	200 + CT scan samples	+	Dice Coef.—91.6%
2D CNN	Jin et al. [10]	970 CT Scan samples	+	Accuracy—94.0% AUC—0.979
SVM + Wavelet transformation	Barstugan et al. [39]	150 CT Scan Samples	+	Accuracy—99.68%
Deep CNN(3D) for classification and U-Net for segmentation	Zheng et al. [46]	500 + Samples	+	AUC-ROC—0.959
DCNN	Heinrich et al. [31]	500 + Samples	+	Dice Coef.—71.0%
CNN-LSTM	Islam et al. [60]	4000 + X-ray Samples	+ + +	AUC—0.992 Sensitivity—99.3% Specificity—98.9%
VGG-19-RNN	Zabirul Islam et al. [59]	6000 + x-ray samples(sample with CoViD, pneumonia and normal cases)	+ + +	Accuracy—99.9% AUC—99.9% Recall -99.8%
Ensemble DCCNs	Singh [1]	6000 + (sample with CoViD, tuberculosis, pneumonia)	+ + +	Accuracy—99.2%

^a^Refer to Abbreviations for detailed nomenclature

There has been active research in biomedical image analysis using deep learning methods, whereby deep learning seems to have outperformed most computer vision problems for instance [8]. Nevertheless, computer vision techniques have shown vast opportunities in numerous application areas, especially in medical research and healthcare [9]. Medical imaging does provide better visibility than standard medical records’ data assessment, such as solving for Diabetic Retinopathy [10]. High-resolution images analyzed can provide any growth details on actuals, on a day-to-day basis, helping a medical practitioner to evaluate the situation quickly and provide a better treatment. It is apparent to be mentioned that the success of leveraging deep learning over traditional machine learning methods have been studied along with wide area of application in the medical domain [11]. Moreover, recent developments of quantum computing, vis a vis its application of quantum algorithm in varied domains, has now opened up new research areas for further optimizing classical machine learning problems [12]. In fact, recently, researchers from Massachusetts Institute of Technology (MIT) created an algorithm to overcome the challenges of developing computationally efficient and performing algorithms in order to solve several medical imaging problems [13].

The domain of medical science needs significant development for making sense of an analysis generated from an image. Previous studies dealing with this topic, have discussed the varied applications of machine learning, deep learning, and quantum algorithms in drug discovery and screening process, thereby solving problems that include compound property and activity prediction, using multitask DNN on 12,000 compounds [14]. Importantly, Quantum is a new paradigm today, with multiple applications being evaluated to solve problems in the fields of optimizing deep learning or machine learning tasks, finance [15], drug discovery [16], along with helping in shedding light on various clinical research [17]. Table 2 enlists extant literature that has dealt with drug discovery.

**Table 2:** Previously studied applications of machine learning in drug discovery and medical diagnosis

Description of study	Author	Methods^a^
Skin cancer detection	Kadampur and Al Riyaee [9]	DNN
Protein structure prediction	Torrisi et al. [18]	DL-CNN, DL-RNN
Cuneiform Dehydration Method for Medical Diagnosis	Baranov [50]	Image Filtering, thresholding, Gaussian blur
Quantitative structure–activity relationship analysis in drug discovery	Uesawa [51]	Deep learning
Quantum chemical properties analysis	Gilmer et al. [52]	Message passing neural network (MPNN)
Predicting compound property and activity	Mayr et al. [27]	Multitask DNN
Predicting pharmacological properties of drugs and for drug repurposing leveraging transcriptomic data from the LINCS project	Aliper et al. [53]	DNN
Automatic molecular structure learning	Merkwirth and Lengauer [54] Lusci et al. [55]	DNN and RNN
Method to model drug induced liver injury (DILI)	Xu et al. [56]	UGRNN
Neural fingerprints of the compound	Duvenaud et al. [57]	Graph CNN
Predicting the ligand–protein interactions	Gomes et al. [48]	CNN, DNN
Predicting the reactions and retrosynthetic analysis	Liu et al. [36]	Neural sequence to sequence model and Monte-Carlo tree search
Drug discovery with on short learning	Altae-Tran et al. [58]	LSTM
Visual Screening from protein–ligand complex	Pereira et al. [49]	DNN
Facilitating probe selection for gene-expression arrays	Tobler et al. [3]	Naïve Bayes, neural nets

^a^Refer to Abbreviations for detailed nomenclature

Although, there have been other studies that have deliberated upon the success of employing deep learning in drug discovery [16] and MRI image analysis for brain tumors, and for detecting and segmenting pneumonia traces using classical machine learning models [6] or leveraging deep learning in biomedical image segmentation applications [18]. The core purpose of this paper is to evaluate and provide empirical evidence for applying Quantum algorithms in medical imaging and drug discovery problems.

## Quantum machine learning

**Fig. 1:**
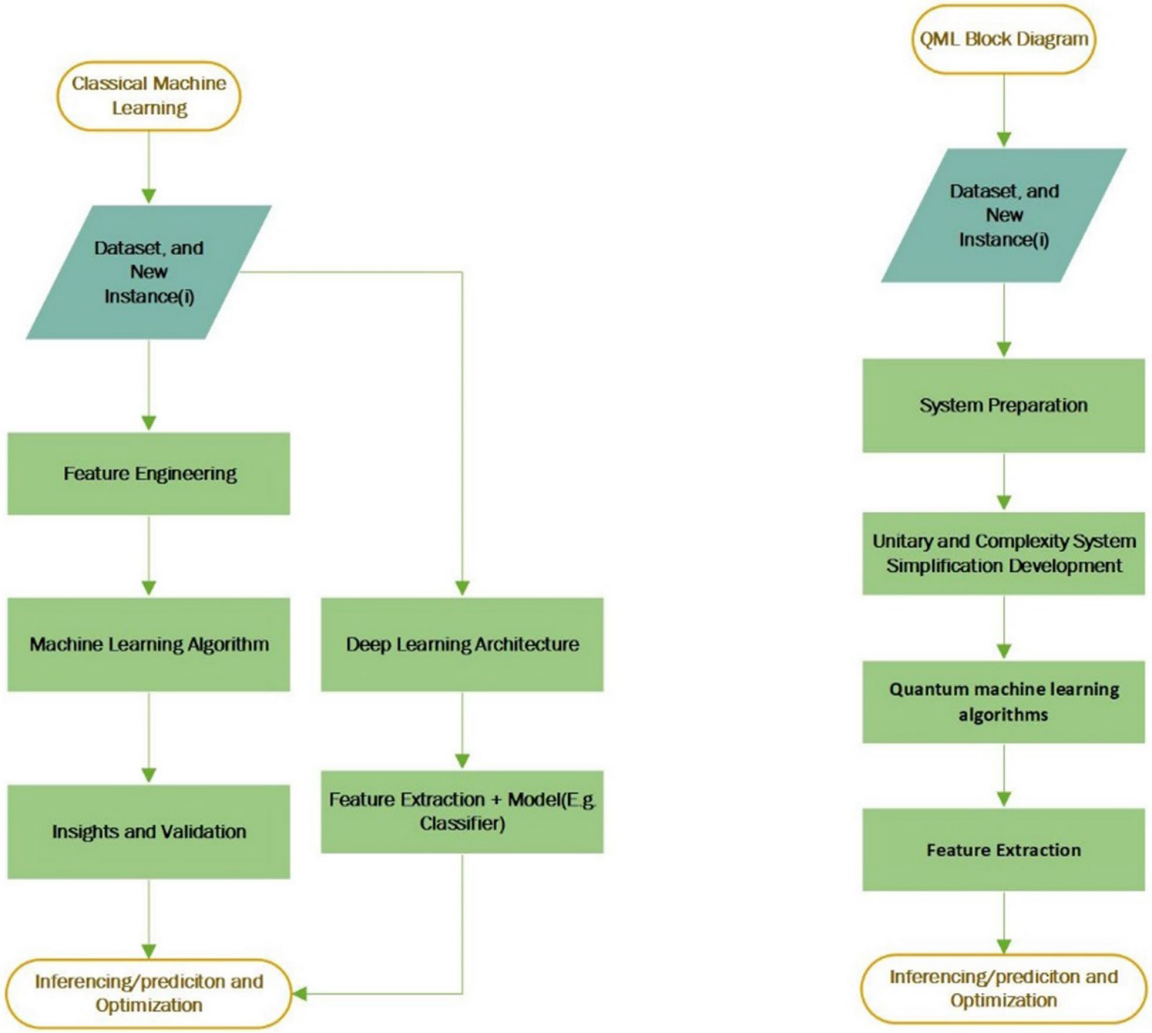
Execution block diagram of classical machine learning/deep learning versus quantum machine learning algorithm designing (refer to Table 4. for algorithmic details on QML)

The recent developments of Quantum Enhanced Learning [19], fusing AI and ML to obtain significant optimal solutions for boosting algorithmic performance has given rise to a new area of research termed ‘Quantum Machine Learning’ (QML), which has effectively evolved from the theory of quantum computing. The concept behind leveraging quantum computing for machine learning tasks is to inherently achieve solution parallelism [20], achieved for optimal constraint solving, using Moore’s law [21]. Quantum algorithms are centered on the concept of Boolean algebra (e.g., OR, AND, and NOT gates) and quantum physics. The data storage layout is established from Quantum bit (Qb) or qubits^1^ that depends on theoretical foundations of electron spin [22]. Importantly, quantum methods in addition can translate other than 1’s or 0’s such as complex information or negative values. A typical model development flow diagram is demonstrated in Fig. 1, explaining the basic control flow difference of classical machine learning versus Quantum machine learning algorithms.

### The need for quantum algorithms

QML is considered as one of the future areas of research in deep learning algorithms. The two key tasks QML can better perform when compared to classical deep learning techniques include:

1. Optimization [23] and Gibbs Sampling [24].
2. Enhance learning algorithms like Bayesian networks [25], Tensors, and search.

QML’s ability to deal with large-scale biased datasets yield faster complexity factors for major classical computing and machine learning tasks, consuming thereby less space and time. Effectively, it uses quantum annealers and tunneling for loss function minimization tasks, solving thereby complex problems of finding super-local minima, and a close approximation of global minima. In fact, multiple methods within the QML strategy are based on fast quantum algorithms for linear algebra, and semi-definitive or constraint-specific problems like optimization, as in the case of neural network models for weight adjustment, during both search and optimization tasks, similar to gradient descent-based optimizer. Assuming the task is to optimize a linear function of MxM matrix(X), over a parallel space with constraints(c), the solver has runtime complexity of O(c(c2 + nω + cns)logO(1)(cnR/ϵ)) [26]. Notably, herein, ϵ is denoted as an approximation factor, while s denotes sparsity, and R represents a range that is bound to yield an optimal matrix(X). Notably, these proven methods outperform classical optimization methods, yielding thereby complexity of O (ncs(Rr/ϵ)4 + ns(Rr/ϵ)7) as proposed by Arora and Kale [27]. The proposed optimizers in turn, tend to improve the overall solution convergence for any machine learning problems.

This paper looks to address two major research questions, while evaluating the application of QML in specific practice, specifically focusing upon medical image diagnostics and/or drug discovery,

#### **RQ1**

Are quantum algorithms suited for large-scale classification problems in medical image diagnostics dealing and industrial applications?

#### **RQ2**

Can quantum algorithms outperform classification or segmentation tasks in comparison with classical deep learning methods w.r.t model efficacy, biased training, and inferencing performance on high-resolution clinical image data?

The research questions would further provide support to exemplify the application of quantum theory in optimizing deep learning techniques to achieve superior performance in solution convergence and quality of the model. Another important aspect to emphasize on supporting production deployment is selecting appropriate quantum hardware for training, while deploying the model for real-time inferencing in health informatics applications, which is discussed later in this paper under the experiment section.

### Application of quantum machine learning

The foundation of QML targets to solve research foundation problems in mathematical analysis to generalize quantum to improve classical learning tasks with potential optimization to speed of execution. Some of the task’s researchers are leveraging includes quantum techniques in Quantum Simulation [28], applied around nanotech, bio-medical imaging, physical chemistry, and with quantum systems tasks, such as search [29], which further provides polynomial speed, as compared to classical algorithms for other varied scenarios.

## Method

The section discusses the methodology followed for building the model, provide details on overall data collection process followed, key modeling process imbibed for the study, and analysis performed with quantum networks.

### Quantum neural network model

**Fig. 2:**
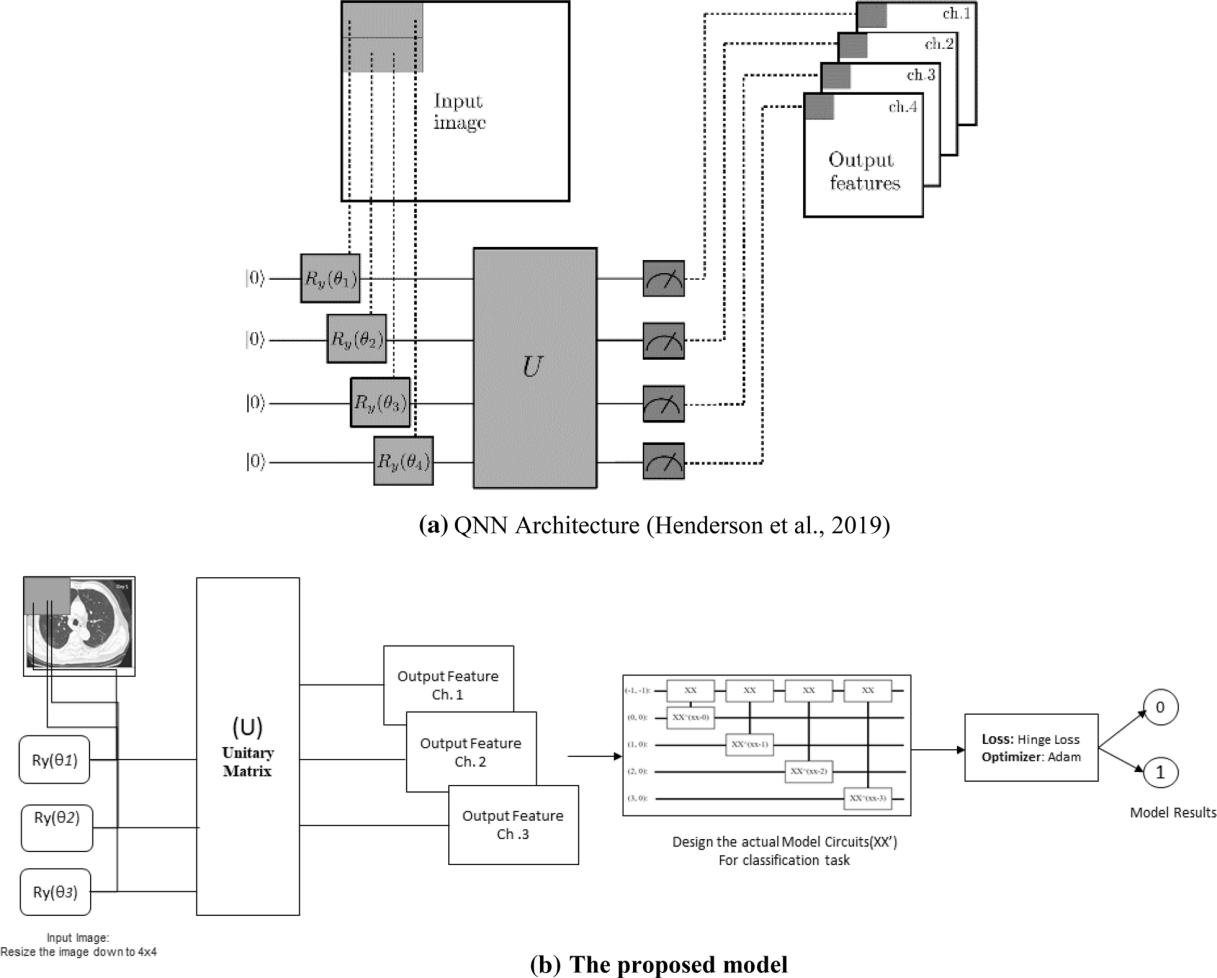
**a** QNN Architecture [31]. **b** The proposed model

This study conducted an experimental analysis with a new variant of a learning model to further take advantage of quantum computing devices to perform learning tasks with quantum data [30]. We assumed that Quanvolutional neural network or Quantum neural network (QNN) would solve classical deep learning problems to be computationally faster from the design paradigm. Figure 2a illustrates the QNN architecture, which would help in understanding the Quantum network design methodology. Further, for simulation, we benchmarked the QNN model across other studies from extant literature. The rationale behind this exercise was to help in exploring varied application scenarios in the medical image analysis task that is presented in subsequent sections. Notably, the process of designing QNN has been described in Table 3 and has been elaborated upon in the subsequent sections.

**Table 3:** Algorithm design stages for quanvolutional neural network

**Fig. 3:**
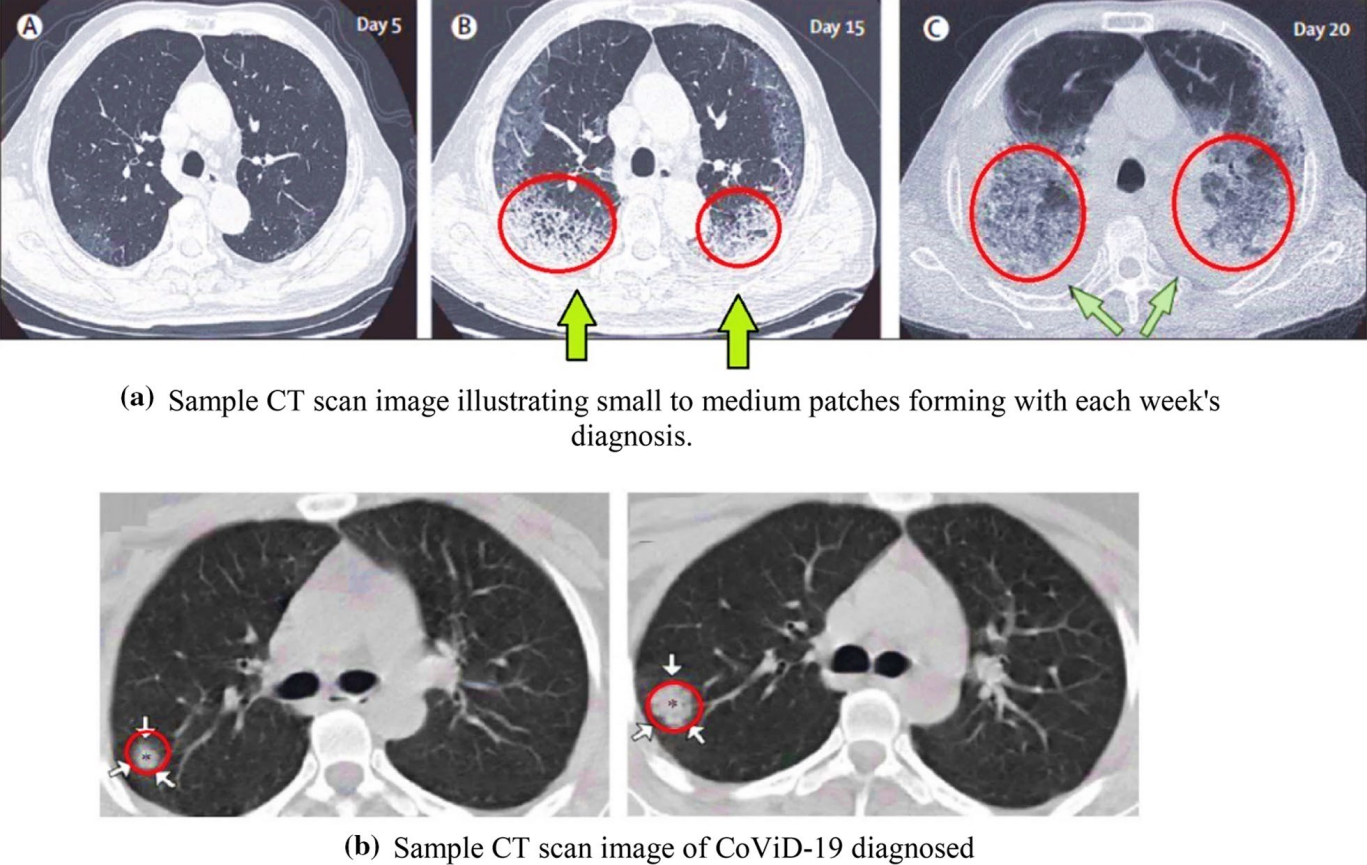
**a** Sample CT scan image illustrating small to medium patches forming with each week's diagnosis. **b** Sample CT scan image of CoViD-19 diagnosed

Stage 1: An input image with small region of interest is embedded into a quantum circuit. An example of a 2 × 22 × 2 square region
Stage 2: A quantum computation, associated with a unitary matrix(U^a^) in Fig. 3, is performed on the system. A Cirq could generate the unitary, most quantum operations have a unitary matrix representation applied to the gate, operations and circuit that represents an object
Stage 3: The system is then quantified by obtaining the list of classical expected values
Stage 4: Similar to the classical convolution layer, each expected value is mapped to a different channel of a single output pixel
Stage 5: The process is iteratively executing across different regions of the image. A full input image scan is viable by re-positioning an output object positioned a multi-channel image
Stage 6: The quantum convolution layer would additionally abide to quantum or classical layers

^a^Refer to Abbreviations for detailed nomenclature

The proposed system is illustrated below in Fig. 2b. From a practical implementation perspective, the model accepts input image rescaled to 4 × 4 size before being fed into the Unitary matrix to obtain features at different channel. Further the features were leveraged to create a quantum circuit model, thereafter, followed by compiling the model using a loss function and optimizer using TensorFlow Keras model utility library.

### Data and pre-processing

This section discusses the data collection process and the pre-processing activities that were conducted during the experiments. Notably herein, the relative transformation measures were required for modeling a QML algorithm. Additionally, this section consists of two sub-sections; the first, describes the data collection process, along with the larger data schema, while the second, discusses the affirmative steps that are taken for pre-processing in pre-modeling stages.

#### Data collection

The model development and verification for quantum deep learning-based image classification would require a large sample set for the quantum machine to perform. As a pre-requisite step, learning models require a significant amount of training dataset for building an efficient model [32], thus a through process was followed for sampling the image files to eliminate any representational biases. Hereafter, this study combined data shared by semanticscholar.com, along with the research work done by Chen et al. [33], and Jin et al. [34].

The data collection process adopted a strategy to collect CT scan samples of varied age groups, ranging from 20–30, 30–45, 45–60, and above 60 years of age, with both positive and negative samples. Key sources were identified, based on represented data statistics (Table 4), along with other open dataset sources from Microsoft open research database, Google dataset search, Stanford, and MIT datasets. Notably, the data search process ensured that the data resolutions were consistent across all the sources. The preferred resolution range of images were chosen (256 × 256 and 448 × 448), because image resolution does play a vital role in deep learning space, and often, high-resolution images do go on to impact model training performance and efficacy to a great extent [35].

**Table 4:** Dataset

Dataset description	Data statistics	Source
CT scans for COVID-19	349 CT images of 216 patients	https://github.com/UCSD-AI4H/COVID-CT
SIRM COVID-19 database	Sample < 50 images	https://www.sirm.org/en/2020/03/31/COVID-19-case-4/
Radiopedia COVID dataset	Sample < 50 images	https://radiopedia.com
Eurorad dataset	Sample < 50 images	https://www.eurorad.org/case/16689
Center for artificial intelligence in medicine and imaging	More than 5000 + sample images of patients	https://aimi.stanford.edu/resources/COVID19#data
Total samples selected	~ 10,000 +	

**Fig. 4:**
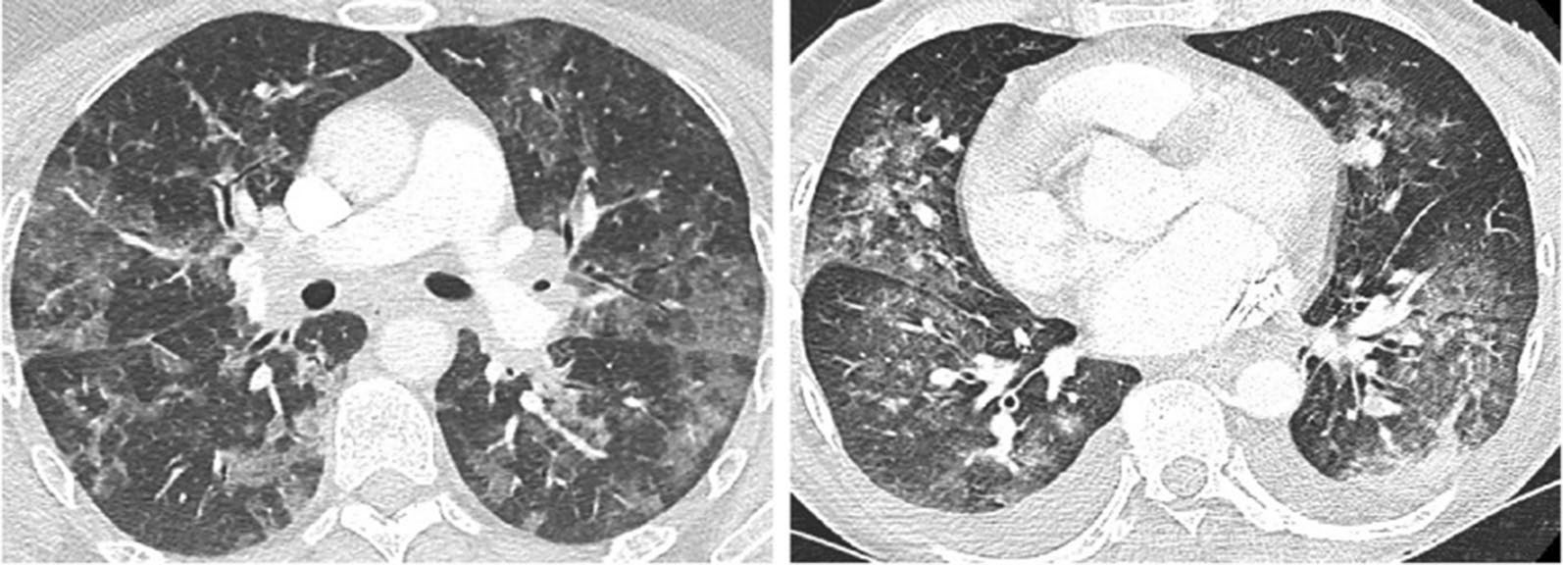
CT scan of two patients of 45-year and 48-years of age with influenza virus pneumonia and Goodpasture syndrome shows bilateral ground-glass opacities in contrast to COVD-19 patients (Hani et al. 2020)

Figures 3a, b and 4, adopted from Shi et al. [36]; Li et al. [37]; and Hani et al. [38] represent classical CoViD-19 and non-COVID-19 (influenzas and virus pneumonia) scans. Based on this, we discuss the overall finding that was observed from CT scans taken through a specific time duration.

The sample data illustrated in Fig. 3a is identified as CoViD-19 positive. A significant growth in building patches was observed in the lungs over 10 days during quarantine (day 5 and day 15 scan). The scan of day 20 showed the formation of a dense mucus that was concentrated across the lungs. Figure 3b illustrates mucus, segmented across a small patch growth across two weeks of supervision. An evaluation dataset of non-CoViD-19 suffering from pneumonia and influenza was also sourced for validation of the model results.

#### Pre-processing and normalization

Importantly, the datasets that were used come from multiple sources; the process of data normalization included comparing the homogeneity of data sources, while further calibrating the images to the required scale for modeling. The study leveraged upon color models of an abstract mathematical model, describing the way colors can effectively be represented as tuples of numbers that are useful in viewing conditions. Once the image was thoroughly analyzed, the dataset was normalized using erosion and dilation [39], leveraging upon OpenCV library, a morphological transformation method, primarily used for handling noise, or detecting intensity collisions. Further, image de-noising (Buades et al. 2011) and scaling was done, using Python-OpenCV library, which in turn, was implemented to the entire dataset for standardization, with the help of fast Nl Means Denoising function for colored images, where the source image input of 8-bit 3-channel images were provided with template window size of 7 pixels and 21-pixel, and hColor of 10 in order to remove the colored noise; post this, they were kept into consideration for the completion of the de-noising process. Notably herein, de-noising generally impacts the image segmentation process in the overall modeling situation.

#### Handling representation and measuring bias in image dataset

The section discusses the impact of measuring bias that depicts the systematic value distortion, which takes place when an issue with a specific device is utilized to visualize and observe an image quality from a training perspective. Importantly, this type of bias is hard to replicate with sampling technique, and thus requires a manual review of the colored images, being used for training [40]. This study further leverages upon bootstrapping resampling technique [41] with different ratios to assemble the required representation of the dataset for experiments.

## Experiments

**Fig. 5:**
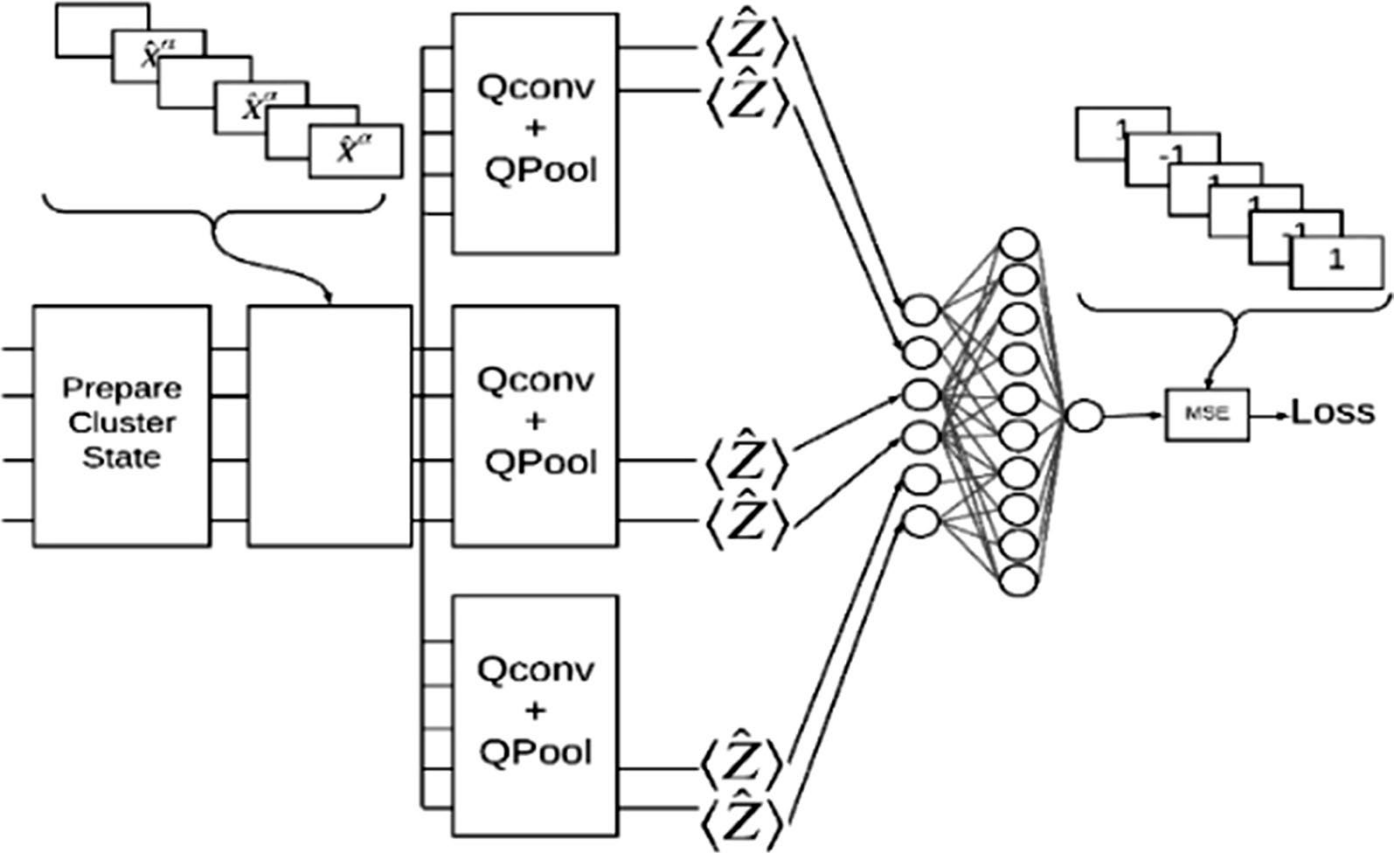
A quantum CNN: hybrid Convolution with multiple quantum filters

In this section, the data pre-processing, model implementation, and evaluation methods have been explained. The experiment processes involved choosing a base model for initial trials and develop the same using the data collected. Based on various performance criteria compared between QNN, QCNN,^2^ Hybrid CNN with a single filter and Hybrid CNN with multiple filters (Fig. 5) from the simplicity of circuit design and performance measurement, QNN was chosen for remaining benchmarking during the trials. Furthermore, the experiments were performed using TensorFlow Quantum (TFQ), and a python framework for QML development. Notably, we leveraged upon D-wave Leap and TensorFlow Quantum Framework as a platform for training and evaluating the experimental setup. The estimated wait time for problem submission was 1–10 s on a 2041 qubits system, under 13.5 qubit temperature (mK). Detailed specifications of the platform may be referred from D-wave and TensorFlow.^3^

**Fig. 6:**
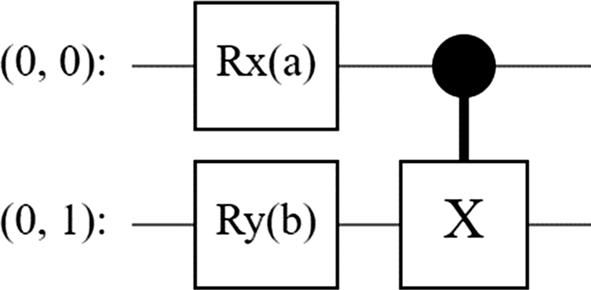
A quantum circuit

TFQ's core focus area is generally on quantum data, and a hybrid quantum-classical model. Various components that need to be followed to build a quantum circuit within the TensorFlow environment have been described below,

- **Circuit**—Cirq is used to design the quantum circuit (Fig. 6). Cirq^4^ is a python framework for writing, optimizing quantum circuits executing in quantum hardware.
- **Pauli Sum**—the linear combinations of tensor products of Pauli operators^5^ defined in Cirq is represented by Pauli sum, operations like circuits, create batches of operators of varying size are of such type.

The experiments performed, involved various feature engineering and transformation stages applied to design. Since the quantum machine cannot handle the large size of the image, we re-scaled it to 4 × 4 at the data processing stage. The overall image calibration steps performed are stated as below,

1. Input raw data using Keras
2. Filtering the dataset to only 3 s and 6 s
3. Downscales the images to fit in a quantum hardware.
4. Treating and removing contradictory examples
5. Convert binary images to Cirq circuits
6. Convert the Cirq circuits to a TensorFlow quantum circuits

**Fig. 7:**
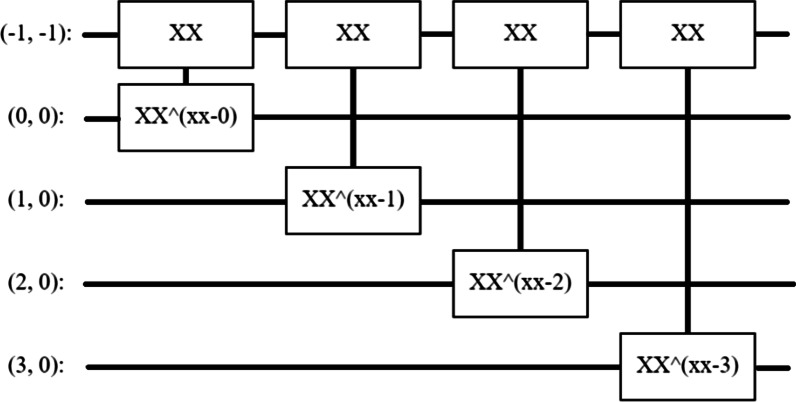
The circuit from the training samples in the first iteration of the 2-layer circuit

In QML, a pixel is represented as a qubit, wherein each stage would actually depend on the pixel value. The process of encoding the data into the Quantum circuit was iterated at multiple threshold values, in the range [0.5, 0.6, 0.7]. A circuit at 0.5 threshold is represented in Fig. 7, and which effectively is a form of 2-layer circuit design for binary classification problems. In terms of model development, various iterations were performed to optimize the general performance of the model through a range of hyper-parametrization testing at various epochs.

Finally, the experiment encompassed creating a 2-layered model (Fig. 7) fitting the data-circuit size, including both preparation and readout operations. In fact, this method could possibly be compared to running a small recurring neural network across pixels. Notably herein, each layer uses *n* instances of the same gate, with each of the data qubits acting on the readout qubit. Additionally, the model building process further used hinge loss as a loss function, along with adaptive learning rate optimization (ADAM) optimizer instead of stochastic gradient descent-based optimizer, which was computationally inexpensive, and possibly even easier to implement. The experimentation process was conducted with different epoch and batch sizes, and the results are presented in the following sections; notably, the overall model parameters are shown in Table 5 below.

**Table 5:** QNN parameter

Parameter(s)	Value
Layer	PQC
Output shape	(None, 1)
Param	32
Model	Sequential
Loss function	Hinge
Optimizer	ADAM
Evaluation metrics	Hinge accuracy

## Evaluation criteria

This paper presents validation loss and validation accuracy as evaluation criteria for the QNN model, whereby the selected metric is ‘hinge loss’ for the experiment, as the problem formulation alludes to a binary classification problem [42]. Importantly, the ‘hinge loss’ represents the difference in prediction from actuals. Moreover, since validation loss is not used to update weights in general, it possibly serves as the right measure of any neural network model. A hinge loss^6^ i.e., l(y) is calculated by comparing prediction (y) with the actual target for prediction (t), followed by subtracting the value from 1, while computing thereafter the maximum value between 0 and the result of the earlier computation.

## Results

To conclude the experimentation process and benchmarking with other relevant methods adopted for detecting COVID-19 patients, the model built, used a 9500-training dataset, encompassing an evaluation conducted over 1500 validation sample sets. The efficacy and the performance evaluation criteria are based on experiments performed with three, five and ten epochs and similar batch sizes. The key metrices taken into the consideration are loss and hinge accuracy shown in Table 6 and confusion matrix shown in Table 7.

**Table 6:** Loss score and hinge accuracy

Epoch	Loss	Hinge accuracy	Validation loss	Val hinge accuracy
1/10	0.6566	0.7534	0.3870	0.8160
2/10	0.3568	0.8263	0.3348	0.8311
3/10	0.3281	0.8497	0.3269	0.8579
4/10	0.2994	0.9061	0.2894	0.8769
5/10	0.2707	0.9542	0.2594	0.8978
6/10	0.2707	0.9582	0.2293	0.9188
7/10	0.2133	0.9586	0.1993	0.9397
8/10	0.1872	0.9582	0.1692	0.9607
9/10	0.1872	0.9582	0.1692	0.9607
10/10	0.1821	0.9692	0.1691	0.9657

**Table 7:**
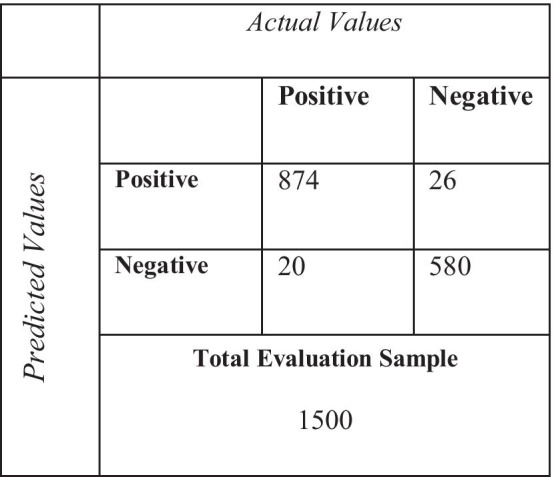
Confusion matrix

**Fig. 8:**
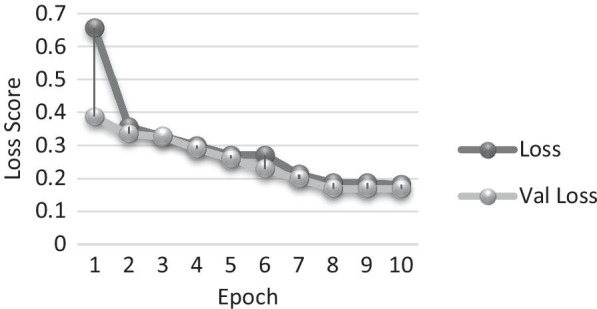
Change in loss per epoch (training and validation)

The change in epoch lowers the loss score, while improving the overall hinge accuracy. The change in accuracy score sees a significant lift after the third epoch, and gradually improves, reducing thereby the *loss* to 0.1559; notably, the percentage score of the change of loss is shown in the analysis in Fig. 8. The overall *precision* of the implemented model is 97.11%, whereas *recall* is 97.76% respectively.

**Fig. 9:**
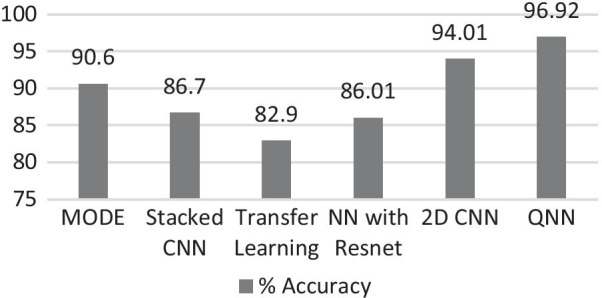
Comparison of various DL models versus QNN

While comparing the traditional deep learning model with QNN (Fig. 9), the latter obtained a 2.92% lift from the 2D CNN model, trained for classification problems in the CT scan data set of COVID-19 patients. The analysis further describes the efficiency of the overall system to scale classification models, whereby the total time to train the model with 9500 images was observed to be 52 min on quantum optimized hardware, while compared to an experiment using CNN on K80 GPU Instance.

## Discussion

The study does entail certain limitations; for instance, the dataset and the training time comparison were limited to available samples. The images collected were limited to CT-Scan with a focus on identifying discoverable patches denoting COVID-19 infection. Further, a detailed study is required to incorporate signals that may occur in a CT-Scan image tending to non-COVID signals to make the model more robust on detection. Further, this study is limited to a minimum viable solution model that would possibly need additional research to take the present version of the model into readily deployable services mode, within the ambit of the biomedical device ecosystem from an end-to-end technology implementation standpoint, supporting thereby large-scale usage in clinical trials.

The crucial point of discussion from a future research standpoint would be around how to leverage the power of quantum algorithms on hardware and localize it to biomedical devices for seamless analysis. This study did demonstrate a substantial advantage to overall medical imaging problems, using quantum learning techniques, while also implementing classical learning models in the context for performance and efficacy in improved model implementations. The model demonstrates robustness while comparing the overall recall value, as any incorrect misclassification of CoViD-19 infected patient could lead to reduction in the overall significances of the predicted outcome further deferring accurate medical diagnosis when compared to a wrongly classified patient with pneumonia or other viral infection that may show similar strains.

While the study also discussed the aspects of hardware requirements for training and evaluation of such models, significant research is still under process, whereby there has been an attempt to launch an economical cloud platform for quantum hardware simulation and modeling. Further, this study provides scope for new development area of edge-quantum computing, and opens up research dialogues around faster diagnostics, and easy interpretation of quantum algorithms in the medical world.

## Conclusion

In this study, we proposed a quantum neural learning model to classify patients with COVID-19 infection, leveraging upon computed tomography scan images in medical diagnosis. The suggested model attained optimal degree of model efficacy during an experimental comparison, yielding 96.92% of accuracy overall, leveraging 9500 + CT-Scan sample images. Additionally, the overall computation time for training the model recorded was 52 min, with the entire sample, along with the inferencing time recorded, which was a minute per image. This overall model training time was significantly less as compared to classical CNN model building with similar samples, using quantum hardware. Our results thereby yield not only a significant lift in the overall accuracy, but also optimizes upon the execution time. The model could be further deployed in clinical trials and medical diagnoses, which have a significant impact on overall decision support for treating patients with early symptoms. Moreover, a medical practitioner could also leverage upon our framework for quicker diagnostics, helping him/her to follow-up with the right treatment, and thereby save a life.

The impact of this paper not only quantifies the ability of QML, but also would help clinical scientists build diagnostic tools applied to drug discovery and disease identification problems with much faster analytical capability using quantum hardware. The study further provides prospects to evaluate quantum algorithms for more complex problems pertaining to image segmentation.

## Data Availability

The datasets generated and/or analyzed during the current study are available in the GitHub repository, https://bit.ly/3xGUS8Q. The dataset does not consist of any confidential data.

## Abbreviations

DL: Deep learning
CNN: Convolutional neural network
QNN: Quantum neural network
QCNN: Quantum convolutional neural network
ADAM: Adaptive learning rate optimization
MODE: Multi-objective differential evolution
RNN: Recurring neural networks
AUC-ROC: Area under the curve-receiver operating curve
DNN: Deep neural network
LSTM: Long short-term memory
CT: Computed tomography
CXR: Chest x-ray
Unitary Matrix(U): In Quantum mechanics, the Hermitian adjoint of a matrix is denoted by a dagger (†) and the equation is represented by U†U = UU† = I, comparable with linear algebra equation U*U = UU* = I, when composite square matrix U is unitary, if conjugate transpose U* is inverse
Pauli Sum: Calculate and simplify the sum of Pauli operators (I, X, Y, Z) according to Pauli algebra rules
Circuit: It represents Quantum model, a sequence of quantum gates for performing a series of computations
Hinge Loss Formula: L(y) l(y) = max(0,1 − t*y)
Qubit: A qubit is a basic unit of quantum information representing information as 0 or 1
CoViD-19: Coronavirus disease

## Footnotes

1 Refer to Abbreviations for detailed nomenclature.

2 Refer to Abbreviations.

3 https://www.dwavesys.com; https://www.tensorflow.org/quantum/concepts

4 Refer to Abbreviations for detailed nomenclature.

5 Refer to definition of pauli operators in nomenclature section.

6 Refer to Abbreviations for detailed nomenclature.
